# Influence of the variation of geometrical and topological traits on light interception efficiency of apple trees: sensitivity analysis and metamodelling for ideotype definition

**DOI:** 10.1093/aob/mcu034

**Published:** 2014-04-10

**Authors:** David Da Silva, Liqi Han, Robert Faivre, Evelyne Costes

**Affiliations:** 1 INRA, UMR 1334 Plant Genetic Improvement and Adaption (AGAP), Montpellier, France; 2 School of Computer Engineering, Weifang University, Weifang, China; 3 INRA, UR 875 Applied Mathematics and Informatics (MIA), Castanet-Tolosan, France

**Keywords:** Silhouette to total area ratio, STAR, functional–structural growth modelling, leaf area, branching, sensitivity analysis, apple, ideotype, *Malus* × *domestica*

## Abstract

**Background and Aims:**

The impact of a fruit tree's architecture on its performance is still under debate, especially with regard to the definition of varietal ideotypes and the selection of architectural traits in breeding programmes. This study aimed at providing proof that a modelling approach can contribute to this debate, by using *in silico* exploration of different combinations of traits and their consequences on light interception, here considered as one of the key parameters to optimize fruit tree production.

**Methods:**

The variability of organ geometrical traits, previously described in a bi-parental population, was used to simulate 1- to 5-year-old apple trees (*Malus* × *domestica*). Branching sequences along trunks observed during the first year of growth of the same hybrid trees were used to initiate the simulations, and hidden semi-Markov chains previously parameterized were used in subsequent years. Tree total leaf area (TLA) and silhouette to total area ratio (STAR) values were estimated, and a sensitivity analysis was performed, based on a metamodelling approach and a generalized additive model (GAM), to analyse the relative impact of organ geometry and lateral shoot types on STAR.

**Key Results:**

A larger increase over years in TLA mean and variance was generated by varying branching along trunks than by varying organ geometry, whereas the inverse was observed for STAR, where mean values stabilized from year 3 to year 5. The internode length and leaf area had the highest impact on STAR, whereas long sylleptic shoots had a more significant effect than proleptic shoots. Although the GAM did not account for interactions, the additive effects of the geometrical factors explained >90% of STAR variation, but much less in the case of branching factors.

**Conclusions:**

This study demonstrates that the proposed modelling approach could contribute to screening architectural traits and their relative impact on tree performance, here viewed through light interception. Even though trait combinations and antagonism will need further investigation, the approach opens up new perspectives for breeding and genetic selection to be assisted by varietal ideotype definition.

## INTRODUCTION

Since the initial proposition by Donald (1968) to develop ideotypes, ‘i.e. biological models which perform or behave in a predictable manner within a defined environment’, a large number of studies have been devoted to their development with a remarkable evolution towards the research of an ‘ideal’ plant for a given context. The concept of ideotype has been particularly developed in crops (Peng *et al*., 2008; Cairns *et al.*, 2012; Chardon *et al.*, 2012) whereas it has remained scarce for trees. In fruit trees, this concept has been explored for apple trees, *Malus* × *domestica*, and several propositions have been formulated for promoting either dwarfed spur types (Dickman *et al*., 1994) or trees characterized by long and weeping branching (Type IV) which were considered prone to regular bearing (Lespinasse, 1992; Laurens *et al.*, 2000). However, the existence of discrepancies between branching types and fruiting regularity was underlined, and further research appeared necessary to improve ideotype definition in this species (Lauri and Costes, 2004). In particular, it was proposed to investigate further the impact of branching along trunks, especially during early tree growth, on tree architecture and performance. The present study aims at contributing to this perspective, by exploring *in silico* different combinations of traits and their consequence on light interception, here considered as an output variable. We expect this study to provide the proof that such an approach is relevant for trees, as previously shown in annual crops (e.g. Brown *et al.*, 2013), and could be applied to other input traits and output tree characteristics.

Among different traits that can be considered to define ideotype depending on the target organs, traits or systems (Andrivon *et al.*, 2013), we concentrate this study on the relationship between tree architecture and light interception. Indeed, tree architecture has a strong impact on light interception, water transport and transpiration, as well as carbon acquisition and allocation. Therefore, the optimization of tree architectures has been considered desirable in fruit tree culture for a long time, in order to improve fruit production in quantity, regularity and quality. Tree manipulations such as grafting, shoot bending and pruning are widely used to modify the tree structure, and represent a constant objective for growers which has resulted in the setting up of numerous training systems (Forshey *et al.*, 1992). From the progressive emergence of a better understanding of tree architectural development and variations among apple tree cultivars (Lespinasse, 1992), alternative training strategies have been proposed which aim at reducing the time spent in orchards for human manipulations of trees (Lauri, 2002). However, a step forward could be made by accounting for tree developmental traits in breeding programmes to select new genotypes with desired properties in terms of tree architecture and light interception efficiency (LIE). For apple trees, most architectural traits, either topological or geometrical, were shown to be genetically controlled, particularly during the first years of tree life (Segura *et al.*, 2008). However, it remains difficult to integrate these traits in breeding programmes due to the complex changes in trait values during tree development (Laurens *et al*., 2000; Segura *et al.*, 2008). In addition, considering the complexity of apple tree architecture, the number of trees for experiments in quantitative genetics, and the long tree growth period covering years, it is neither convenient to grow and then measure phenotypes in the field nor easy to collect data. Moreover, there are few methods to quantify and compare in an objective way the impact of training systems or cultivars on LIE. One possible approach is to compare three-dimensional (3-D) representations of foliage geometry, using either an electromagnetic 3-D digitizer (Sinoquet and Rivet, 1997; Massonnet *et al.*, 2008; Saudreau *et al.*, 2011) or 3-D reconstruction from terrestrial light detection and ranging (LiDAR) systems (Côté *et al.*, 2009; Livny *et al.*, 2010; Preuksakarn *et al.*, 2010). However, on the one hand, 3-D digitizing does not allow the description of trees over time and the number of described trees remains low because it relies on difficulat and time-consuming field measurements. On the other hand, reconstruction of geometry and topology from LiDAR-generated data is an actual research topic that still poses some challenges but is a promising alternative that could overcome the restriction of magnetic digitizing (Raumonen *et al.*, 2013). To save time, labour and resources, we thus initiated a strategy based on computer-based virtual experiments to explore the impact of traits variations on LIE over years.

Among geometrical traits, the choice of an individual leaf area was dictated by its major role, in conjunction with their within-tree 3-D distribution, in light interception (Sinoquet *et al.*, 1991) and by their highly heritable values in different crops (Byrne *et al.*, 1996; Richards *et al.*, 2002). In the apple tree, total leaf area and leaf area per shoot types have been considered as key variables in light interception and fruit production (Wünsche *et al.*, 2000). Moreover, individual leaf area is a heritable trait in the apple tree, as far as it is considered on a homogeneous shoot type (pers. obs.). Internode dimensions also have large effects on plant properties, in particular on leaf spatial distribution and LIE (Dechaine *et al.*, 2007; Bell and Galloway, 2008), and for disease propagation (Le May *et al.*, 2009). From an evolutionary perspective, internode elongation is considered as having an adaptive value since its variation in response to environmental changes provides a fitness benefit to the species (van Kleunen and Fischer, 2004; Thomas and Hay, 2008). Moreover, intraspecific variation of internode length has been exploited in agronomy, or even controlled through plant regulator application, for controlling plant size and improving yields, particularly in grain crops (Khush, 2001; Salamini, 2003). Even though it is influenced by both environmental and ontogenic factors, internode length is under a strong genetic control in the apple tree (Segura *et al.*, 2007, 2009), thus opening up new perspectives for controlling fruit tree size through breeding programmes. Two additional geometrical traits were considered in our approach, the shoot apical diameter and branching angles. Shoot and trunk diameters are highly heritable in different forest (Costa and Durel, 1996) or fruit trees, including apple (Tancred *et al*., 1995; Durel *et al*., 1998; Oraguzie *et al*., 2001). Among different diameters that can be measured within apple tree crowns, the shoot top diameter was chosen because it exhibited a stronger genetic effect than the basal diameter (Segura *et al.*, 2008). This is probably because the top diameter does not integrate secondary growth because the measured stem segment is just below the shoot apex and it is not a cumulated variable over time as is basal diameter. Moreover, shoot diameter has a main impact on shoot bending (Alméras *et al.*, 2002, 2004). More precisely, the slenderness, i.e. the ratio between the length and diameter, appeared to be the main factor involved in shoot bending. Thus, by considering a range of values for both these variables, we expected to generate a range of tree architectures which would differ in shoot bending and therefore in leaf spatial distribution.

The second category of variables we considered in this study regards tree topology. We chose to focus on branching traits along the first annual shoots of the trunk, which corresponds to an early developmental stage. This choice was dictated by the existence of ontogenetic gradients during tree development (Barthélémy and Caraglio, 2007) which are strongly expressed and rapid in the apple tree (Costes *et al.*, 2003; Segura *et al.*, 2008). Also, the numbers of internodes per growth unit (GU) or axis exhibited lower heritability values or were less dependent on genetic factors than branching variables, even though the two variables were correlated (Segura *et al.*, 2006, 2008). Because, in grafted apple trees, the first years of trunk development usually correspond to the longest annual shoots (Costes *et al.*, 2003), branching along these first annual shoots of the trunks exhibit the most complex patterns within the trees, when compared with other annual shoots sampled at higher branching orders and in older parts of the trees (Renton *et al.*, 2006). These annual shoots bear different kinds of axillary productions which can be categorized depending on their time of outgrowth and fate. In previous studies, axillary productions were classified as latent buds, sylleptic shoots (i.e. which develop immediately without any resting period of the axillary bud) and proleptic shoots (i.e. developed after a winter resting period of the buds). In this last category, three additional classes were considered: flowering shoots, and short and long vegetative shoots. Even though the number of sylleptic laterals is highly heritable in the first year of tree growth and they contribute strongly to the construction of tree shape, this shoot category is rare in the following years (Segura *et al.*, 2008) and is usually assumed to be more involved in tree plasticity in response to environmental conditions than proleptic shoots (Wu and Hinckley, 2001). In contrast, proleptic shoots, especially long ones, are the basis of within-tree repetitions and similarities which is the fundamental rule of tree architectural development (Renton *et al.*, 2006). Their number and location along their bearer shoots are thus assumed to contribute greatly to the within-tree architectural organization and leaf distribution, which in turn determine light interception.

In the present study, we aimed at exploring *in silico* different combinations of organ geometrical and branching traits and their consequence on light interception, here considered as an output variable. We developed a strategy which made use of several models, methods and information already available from previous studies and combined them into three steps: (1) a number of geometrical and topological traits whose range of variation was previously observed within a segregating population of apple hybrids were considered as input parameters in an apple tree architectural model; (2) the 3-D representations resulting from this model were used by an environmental simulation tool to calculate light interception; and (3) a sensitivity analysis was performed on an output variable considered representative of LIE. This strategy allowed us to highlight a hierarchy among the studied factors.

## MATERIALS AND METHODS

For simulating apple tree (*Malus* × *domestica*) topology and geometry over years, we used the architectural model MAppleT, i.e. Markov Apple Tree, which was parameterized for the Fuji cultivar (Costes *et al.*, 2008). The simulated tree topology was tested through comparisons of GU counts per tree and years with observed data on digitized trees. The light interception of the simulated trees was estimated using MμSLIM, namely the Multi-Scale Light Interception Model, which is a model capable of combining a detailed and statistical description of foliage at different scales to estimate radiation attenuation (Da Silva *et al.*, 2008). The tree total leaf area (TLA) and silhouette to total area ratio (STAR) estimated from the combination of MAppleT and MμSLIM were compared with those of digitized trees of the same cultivar (Fuji) and with independent published data on different cultivars (Da Silva *et al*., 2014).

### MAppleT model

MAppleT is an architectural model developed for simulation of apple tree topology and geometry (Costes *et al.*, 2008). In MAppleT, tree topology is organized by Markovian sub-models previously estimated on a collected data set. These sub-models control both the branching pattern and the GU successions along axes (Guédon *et al.*, 2001; Costes and Guédon, 2002, 2012; Costes *et al.*, 2003, 2008; Renton *et al.*, 2006). At the macro scale, the GUs are represented by four states, ‘long’,‘medium’, ‘short’ and ‘floral’, whose succession is modelled by a Markov chain. At the phytomer scale, the branching structures of long and medium GUs are characterized with zones, each zone being characterized by the mixture of laterals its contains. The succession of these zones is modelled with a hidden semi-Markov chain (HSMC) which parameters depend on the parent shoot length (Renton *et al.*, 2006).

For geometrical development, the individual leaf area (LA) development follows a logistic growth curve according to:

(1) $$\text{LA = }f_{l}(d)\;{\text{LA}}_{\max}\left[1-\delta_{\mathrm{rk}}\left(1-\frac{\text{rk}}{n_{p}}\right)\right]$$

where LA_max_ is the maximum leaf area, *f*_l_(*d*) is a normalized logistic function going from 0 to 1 over a period of 12 d of leaf development (E. Costes *et al.*, unpubl. res.), rk is the leaf rank, *n*_p_ is the number of pre-formed leaves and *δ*_rk_ is a Kronecker delta function such that *δ*_rk_ = 1 if rk < *n*_p_, and *δ*_rk_ = 0 otherwise. Thus, the final area of pre-formed leaves depends on their rank. The individual internode length (IL) follows a similar growth over its developmental period (10 d; E. Costes *et al.*, unpubl. res.), reaching a final length that is indexed on the branching zone as follows:

(2) $$\text{IL}={\text{IL}}_{\min}+g_{l}(d){\text{IL}}_{\max}c_{z}$$

where IL_min_ and IL_max_ are the minimum and maximum internode length, respectively, *g*_l_(*d*) is a normalized logistic function going from 0 to 1 over the developmental period and *c*_z_ is a coefficient bounded in [0, 1] that depends on the branching zone.

The MAppleT model is able to simulate the bending of branches through a biomechanical sub-model (Alméras, 2001; Taylor-Hell, 2005). To model branch bending, the torques imposed by gravity and phototropism are applied to each internode and then recursively extended to the entire axis. The resulting flexion depends on the branching angle and the wood elasticity, which in turn depends on the internode diameter. The simulation of cambial growth is supported by the pipe model (Shinozaki *et al.*, 1964), thereby the internode widths are eventually accumulated from the diameters of the corresponding distal ends (including the apical meristems and the leaf petioles).

MAppleT was developed using LPy, a python implementation of the L-System paradigm (Boudon *et al.*, 2012), and is therefore fully integrated in the OpenAlea platform for plant modelling (Pradal *et al.*, 2008). This integration provides a set of tools dedicated to plant modelling, a user-friendly environment, advanced deployment methods and allows easy interactions with other models, e.g. light interception models, through defined input and output interfaces. However, these advantages came at the cost of an increased computational time that can vary, for a 5-year-old simulated tree, from 1 to 36 h depending on the branching density as computational time is directly related to the number of simulated components, i.e. metamers, leaves and fruits. Consequently, to obtain a high number of simulated trees, parallel simulations were implemented using a cluster of computers where each processor was carrying out an entire simulation, i.e. five consecutive years of simulated tree growth.

### Light interception

For evaluation of LIE, the STAR, i.e. the ratio of silhouette area to total leaf area (Oker-Blom and Smolander, 1988; Stenberg, 1996), was used. Silhouette area is the projected area of an object on a plane that is perpendicular to the projective direction. Based on the total area of all leaves and the silhouette area of the tree crown, the STAR for a whole tree can be calculated by:

(3) STAR=PLA/TLA

where PLA is the total projected leaf area, i.e. the silhouette area of the tree (considering overlap between leaves), and TLA is the total leaf area, i.e. $\text{TLA = }\sum_{i=1}^{n}A_{i}$, where *A_i_* is the area of leaf *i* and *n* is the total number of leaves.

The STAR describes directional light interception; thus, in order to obtain sky-integrated values of light interception capacity, the diffuse mode available in MμSLIM was used to simulate the radiance of an overcast sky. The sky hemisphere is discretized in 46 solid angle sectors of equal area according to the Turtle sky proposed by Den Dulk (1989). The directions used are the central direction of each solid angle and the 46 directional STAR values which are summed up with a weighting coefficient derived from the standard overcast sky radiance distribution (Moon and Spencer, 1942). All STAR values hereafter are integrated values over the entire sky dome. The sky-integrated STAR is a good descriptor of LIE in the specific case of a single standing tree, i.e. no community effect, that was chosen in order to focus on the phenotype variability of individual trees.

### Sensitivity analysis

Because of the relatively long time required to run each simulation, it was not possible to carry out in a reasonable time a sensitivity analysis based on eFAST or Sobol methods that would require a large number of simulations (Saltelli *et al.*, 2000; Faivre *et al.*, 2013). For example, to estimate Sobol's sensitivity indices on a tree growth model, Wu *et al*. (2012) needed thousands of model evaluations using Monte Carlo sampling. As our model MAppleT is stochastic, Morris' methodology (which usually is a low-demand screening method for deterministic models) needs a larger set of simulations in order to take stochasticity into account (Degasperi and Gilmore, 2008). Therefore, we adopted a metamodelling approach (or response surface modelling) to investigate our model response on a restricted set of model runs. Among metamodelling approaches (Storlie and Helton, 2008; Villa-Vialaneix *et al.*, 2012), we selected the generalized additive model (GAM) (Wood, 2006) and modelled STAR as an additive sum of non-parametric functions of each input parameter:

(4) $${\text{STAR}}_{i}=f_{0}+\Sigma_{k}\;f_{k}(X_{k,i})+\epsilon_{i}$$

where *k* is the number of parameters *X* for tree *i* and *ϵ_i_* is its residual error term, with the constraints that all the functions *f_k_* have an integrand equal to 0. The residual error term *ϵ_i_* corresponds to the modelling error plus the stochastic effect due to the Markovian random process included in the MAppleT model. The functions considered here correspond to the default option in the mgcv library in R software version 2.13.1, i.e. thin plate regression splines. This modelling permits exploration of the space of parameters without replicates (as in Lurette *et al*., 2009). Also, this model was selected for its robustness and its ability to consider the main effect of each parameter with a non-linear form which is not only polynomial. It provides a visualization of possible deformations of the output variable response with respect to a polynomial model. In the present application of GAM, we did not consider interactions between the input parameters because of the limited size of the data set. We expect such a design to allow us to identify the main effect of each factor, especially the expectation of the response variable Y (here the STAR) along the X input parameter domain (i.e. E(Y|X)).

The estimated response function *f*_k_(*X*_k_) was thus visualized for each input parameter, and characterized by two statistical descriptors, e.d.f. and *F*, which correspond respectively to the equivalent degree of freedom (Woods, 2006) and the *F* statistic for testing if the function *f_k_* is significantly different or not from the null function. The value of the e.d.f., which is an indicator of the non-regularity of the function, is equal to 1 if the function is a perfect line, and to 2 if it is a perfect quadratic curve. When the response function exhibits a positive (vs. negative) slope, it is interpreted as an increase (vs. a decrease) of the variable response with the increase of the input parameter. The intersection of the curve with the *x*-axis, which corresponds to the input parameter values, is obtained when the expected response corresponds to the mean value of the variable response. Hence, when the curve is below the *x*-axis the response will be below the mean for these input values, whereas when the curve is above the *x*-axis, the response will be above the mean.

### Virtual experiment

In the present study, the trait variations for either geometrical or topological traits were extracted from previous observations of apple bi-parental progeny, which were derived from a ‘Starkrimson’ × ‘Granny Smith’ cross, with parents being chosen for their contrasting architecture. The ‘Starkrimson’ maternal parent displayed an erect growth habit with many short shoots and a tendency towards irregular bearing (Lespinasse, 1992). In contrast, the ‘Granny Smith’ pollen parent displayed a weeping habit with long shoots and fruit-bearing regularity. These F_1_ progeny comprised 125 seedlings that were grown on their own roots in a nursery before being grafted on M_9_ rootstock.

#### Geometry

**Fig. 1.**
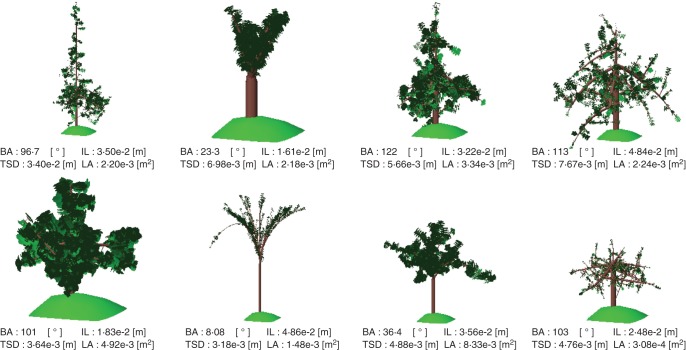
Visualization of simulated architectures for apple trees (*Malus* × *domestica*). These sample architectures were produced by MAppleT with different values of geometrical investigated parameters. The green area below the simulated tree has a constant size and thus its purpose is to act as a visual aid in terms of relative tree size.

Three geometrical aspects were expected to have a direct influence on the STAR value of the whole tree: the leaf surface, the density of leaves and the leaf orientations. The interception surface is mainly determined by the area of an individual leaf. Petiole angle is considered constant in the present version of MAppleT; hence, the leaf orientation is mainly influenced by branching angle and branch bending. The latter depends, for a given wood elasticity, on the allocation of weights imposed by leaves and internodes along an axis. According to the pipe model, the internode widths are recursively accumulated from the diameters of corresponding distal ends, from the shoot top to the shoot base. Therefore, the top shoot diameter is also expected to have an impact on branch bending and, consequently, on leaf orientation. The leaf density is determined by both the intervals between leaves, as determined by internode length, and the branching behaviour of the canopy. Consequently, we chose four geometrical traits related to these aspects, LA, IL, top shoot diameter (TSD) and branching angle (BA), to investigate their complex influences on the whole tree's STAR value. We ranged the four geometrical traits from a lower value to an upper value corresponding to the range of variation observed in the previously studied apple progeny (Segura *et al.*, 2008) (Table 1). This range is larger than that observed in the most commonly used varieties, as exemplified by a segregating population compared with their parents for internode length (Ripetti *et al*., 2008) or branching angles which have been described as varying between 0 (vertical) for upright varieties (e.g. ‘Braeburn’) to 60° for spread varieties (e.g. ‘Reinette Blanche du Canada’; Lespinasse, 1977). The experimental design for exploring the space of input parameter values was performed with LHS (Latin hypercube sample) where the distribution chosen for each input parameter was the non-informative one, with the corresponding procedure in the package lhs of R software version 2·13·1. For this experiment, a sample of 300 sets of parameters was generated. Each set of parameters was then used to simulate the growth of an apple tree over 5 years. All other parameters were kept with default values (Costes *et al.*, 2008). Samples of these simulated tree architectures are shown in Fig. 1. The LIE was estimated at the whole-tree level, on June 30 of each year. This date corresponds to the time when growth stops for most of the shoots, in particular the short and the medium shoots.

**Table 1.** Range of values of the investigated geometrical traits

Parameter	Lower value	Upper value
Leaf area (m^2^)	3·0 × 10^–4^	9·0 × 10^–3^
Internode length (m)	8·0 × 10^–3^	5·0 × 10^–2^
Shoot top diameter (m)	1·0 × 10^–3^	8·5 × 10^–3^
Branching angle (°)	0·0	1·3 × 10^–2^

#### Topology

**Fig. 2.**
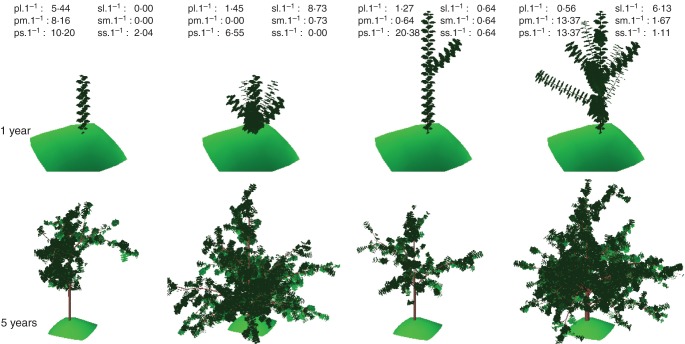
Visualization of simulated architectures for apple trees (*Malus* × *domestica*). These sample architectures were produced by MAppleT with different branching sequences for the trunk first annual shoots. The green area below the simulated tree has a constant size and thus its purpose is to act as a visual aid in terms of relative tree size.

To investigate the effect of the branching during early growth, tree simulations were initiated with sequences of lateral types along the first year of growth of the trunks previously observed (Segura *et al.*, 2008). This allowed us to vary the number of lateral shoots along the trunks, keeping the overall organization of branching. Different axillary shoot types were considered: each axillary bud could develop immediately into a sylleptic shoot or remain latent until the next budbreak when they could develop into proleptic shoots, or still remain latent. Both sylleptic and proleptic shoots could be short, medium or long (for details see Segura *et al.*, 2008). Among the 125 progeny, only 108 recorded trunk sequences were available due to tree-dying and trunk-breaking events. From these sequences, the number of sylleptic and proleptic shoots in each category was considered and divided by the annual trunk length, in order to express a branching density along the trunks. These densities were additionally decomposed according to the shoot length, yielding a maximum of six input parameters: densities for short, medium and long proleptic and sylleptic shoots. Each sequence was then used to initiate the model simulations. All the other parameters of the model were kept with default values (Costes *et al*., 2008). In particular, the Markov models estimated for the Fuji cultivar were used to simulate the type of growth unit and axillary shoots developed from each bud, either terminal or axillary, in the subsequent years. The simulated trees thus differed because of the different initial 1-year-old branching sequences, observed for each hybrid, and the stochasticity of Markovian models in the subsequent years. A sample of initial (1-year-old) and resulting final (5-year-old) simulated tree architectures is shown in Fig. 2. Similarly to the geometrical counterpart of this virtual experiment, the topological part covered 5 years of tree growth and the STAR was calculated at the whole-tree level on 30 June of each year.

## RESULTS

### STAR evolution over years

**Fig. 3.**
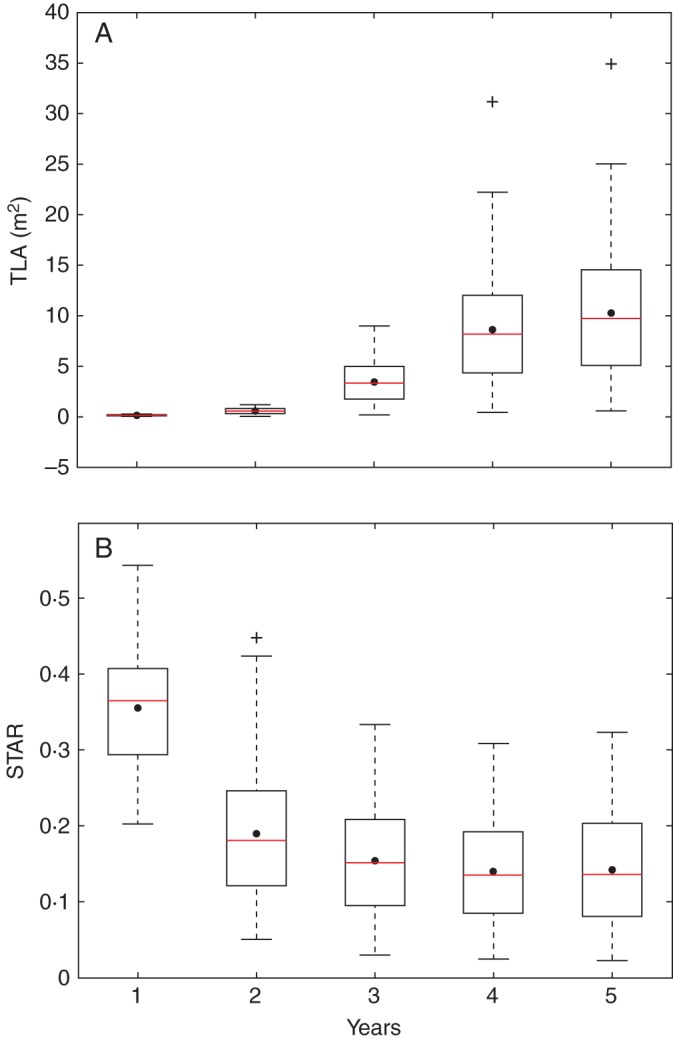
Evolution of TLA (total leaf area) and silhouette to total area ratio (STAR) for 300 simulated trees from the second to fifth year of growth in the geometry experiment, when varying four organ geometry parameters. Each box extends from the lower to upper quartile values, with a red line at the median and a dot at the mean values, respectively.

**Fig. 4.**
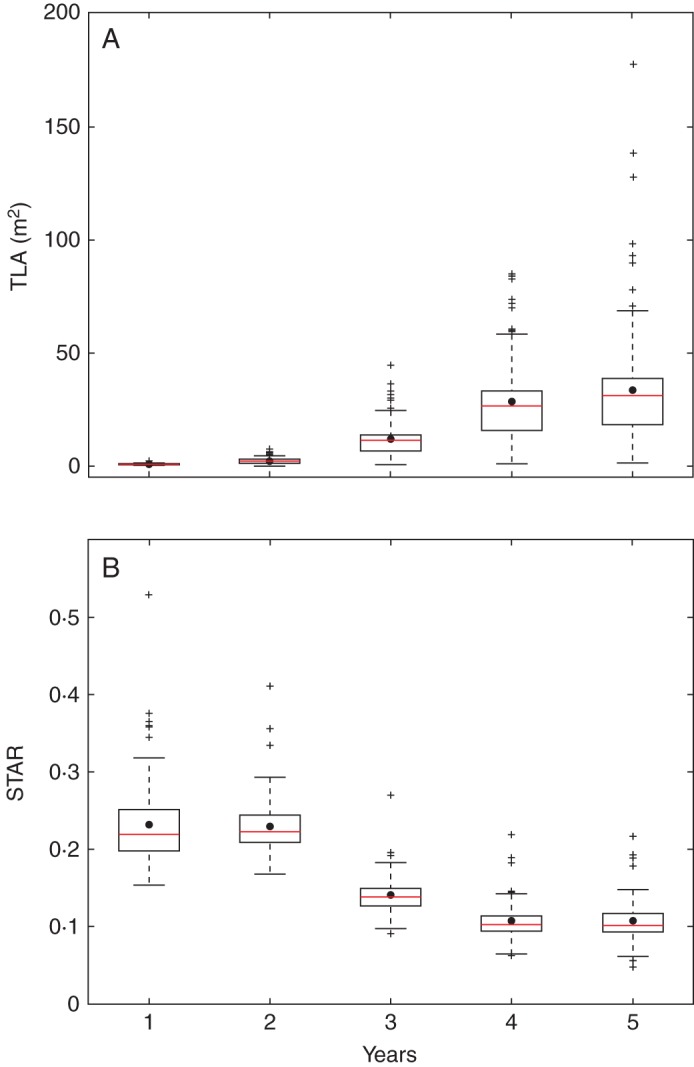
Evolution of TLA (total leaf area) and silhouette to total area ratio (STAR) for 108 simulated trees from the second to fifth year of growth in the topology experiment, when varying the branching sequences of trunk first annual shoots. Each box extends from the lower to upper quartile values, with a red line at the median and a dot at the mean values, respectively.

The distribution of the TLA of the simulated trees was represented from the first to the fifth year of growth for both experiments (Figs 3A and 4A). The mean (represented by dots) and variance (extent of the box) of TLA obtained with the geometry experiment increased rapidly with years. The mean values increased from 0·096 m^2^ in year 1 and to 10·21 m^2^ in year 5. The variability also increased with age, the standard deviation (s.d.) going from 0·052 in year 1 to 6·083 in year 5. However, these variations, which were very rapid from year 1 to year 4, were attenuated between year 4 and year 5. In the topology experiment, the TLA and its s.d. followed the same pattern, with an increase from year 1 to year 5, slightly attenuated between years 4 and 5. The mean and s.d. values increased from 0·69 m^2^ to 33·98 m^2^ and from 0·49 to 26·47, respectively. The mean TLA values and their s.d. were much higher in the topology experiment than in the geometry experiment. This difference was set from the first year of growth with a TLA mean value 5–6 times higher in the topology experiment than in the geometry experiment. This ratio was reduced to 3 when TLA mean values were compared between experiments in year 5. Also, the topology experiment yielded more outlier individuals than the geometry experiment.

In contrast, the mean STAR values, regardless of the experiment and input parameters, decreased with tree age and stabilized when the trees were 4 and 5 years old (Figs 3B and 4B). The mean STAR values from the geometry experiment went from 0·36 in year 1 to 0·14 in year 5, with a variability that remained constant through the years with a s.d. around 0·07 (Fig. 3B). In the case of the topology experiment, the mean STAR values decreased from 0·23 in year 1 to 0·11 in year 5 (Fig. 4B). In this experiment, the s.d. also declined from 0·054 to 0·025 and was significantly lower than in the geometry experiment. Similarly to TLA, differences in STAR mean values were observed from the first simulated year of growth, with higher mean values in the geometry experiment than in the topology experiment. In contrast to TLA, the mean STAR values were lower in the topology experiment than in the geometry experiment, but to a lesser degree than their variability.

### Sensitivity analysis

#### Geometry

The prediction of the STAR value with the GAM model for a specific combination of the four input parameter values is the sum of four terms, corresponding to their respective effects. The approximate effects of the four input parameters on STAR values revealed significant effects of IL, LA and BA in all years, whereas TSD exhibited a non-significant effect, except for year 1 (Table 2). In that year, the BA effect was not significant, and even though TSD had a significant effect, the comparison of the *F*-values showed the overwhelming effect of both IL and LA. This relative effect order, corresponding to decreasing *F*-values, was maintained over the 5 years, with IL having the highest effect, followed by LA, and finally with a moderate effect of BA. The estimated GAM explained from 99·5% in year 1 to 94·0% in year 5 of the total variation of STAR values obtained from simulations (Table 2, adjusted *R*^2^).

**Table 2.** Approximate effects of the geometrical input parameters on STAR values with a GAM

Year	Input variable	e.d.f.	*F*	*P*-value	*R*^2^
1	BA	1	0·09	0·76	0·995
	IL	4·68	6089·98	<2 × 10^–16^***	
	TSD	1·34	19·72	1·65 × 10^–7^***	
	LA	7·33	2542·99	<2 × 10^–16^***	
2	BA	4·58	131·11	<2 × 10^–16^***	0·953
	IL	3·53	642·1	<2 × 10^–16^***	
	TSD	1·14	0·11	0·796	
	LA	6·68	267·05	<2 × 10^–16^***	
3	BA	4·18	142·82	<2 × 10^–16^***	0·955
	IL	2·25	1043·3	<2 × 10^–16^***	
	TSD	1·51	2·1	0·128	
	LA	7·26	268·37	<2 × 10^–16^***	
4	BA	3·7	92·86	<2 × 10^–16^***	0·946
	IL	3·23	595·94	<2 × 10^–16^***	
	TSD	1·35	0·81	0·425	
	LA	8·2	228·4	<2 × 10^–16^***	
5	BA	3·46	92·17	<2 × 10^–16^***	0·94
	IL	3·5	500·71	<2 × 10^–16^***	
	TSD	1·44	1·2	0·299	
	LA	7·49	207·19	<2 × 10^–16^***	

Input variables were the branching angle (BA), the internode length (IL), the top shoot diameter (TSD) and leaf area (LA).

*** *P* ≤ 0·001.

**Fig. 5.**
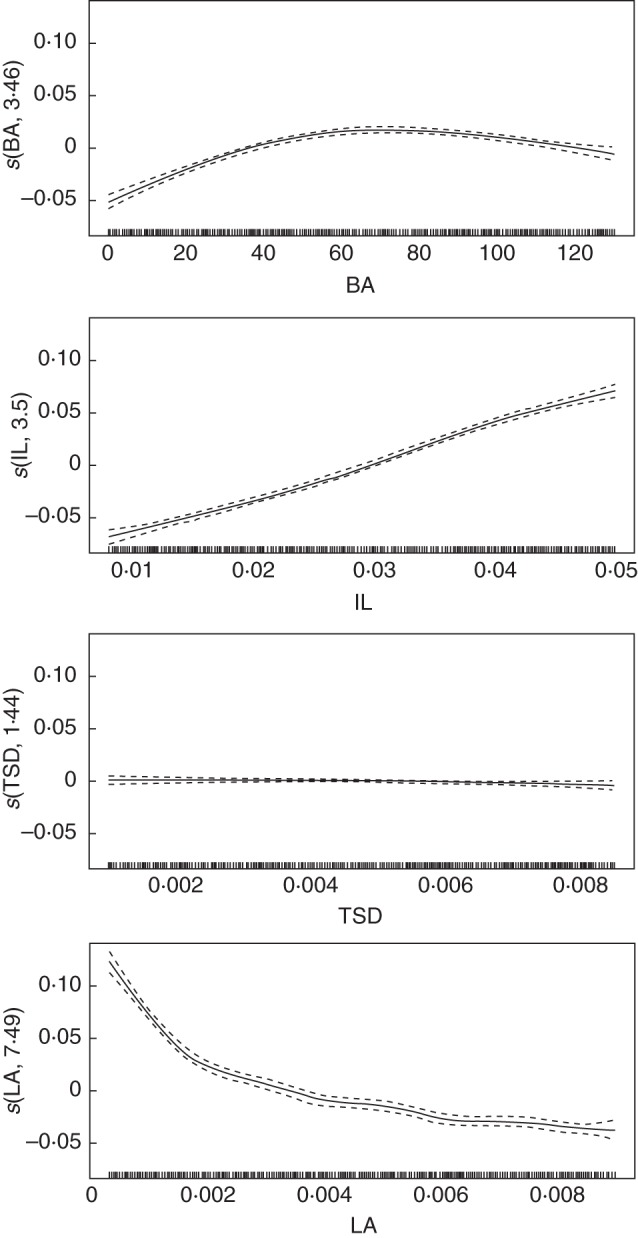
Expected mean values (solid lines) and confidence band (dashed lines) of smoothed terms (*y*-axes; predicted STAR value) depending on the input parameter values in the geometry experiment (*x*-axes: position of sampled values in LHS) estimated from the generalized additive model (GAM). The term *s* in the labels on the *y*-axes indicates that thin plate splines are used, and the number corresponds to the equivalent degree of freedom (e.d.f.) of the estimated curve.

Fluctuations in the shape of the output variable responses are revealed by the value of the e.d.f. (see values in Table 2). As shown by the e.d.f. values, the non-regularity of the functions did not change much over the years. The high impact of IL and LA can be illustrated by the response functions of the expected mean STAR value, illustrated for year 5 in Fig. 5. In year 5, the response function to IL was a monotonous ascending line, slightly sigmoidal, denoting an almost constant positive effect of IL increase on the mean STAR value. This function exhibited a shape similar to a rarefaction curve in year 1 and changed toward a sigmoid shape in the following years by passing through a monotonous ascending line shape in year 3 when the e.d.f. reached its minimum (data not shown). In contrast, the increase in LA had a negative impact on the expected mean STAR value (Fig. 5). This negative effect was stronger up to 0·002 than between 0·002 and 0·008, displaying a curve with a shape reminiscent of a negative exponential. This shape was similar in the previous years (data not shown). LA was the input variable that yielded the more irregular response function, with e.d.f. values between 6·68 and 8·20, significantly higher than those from other input variables. The functions of response to BA exhibited a positive effect from 0 to 65, slowly decreasing with BA increase before the function reached an inflexion point and started to exhibit a low but increasingly negative effect on STAR from 65 onward. The shape of the response function to BA was very different in year 1, where it was a straight horizontal line (denoting no effect at that stage, data not shown). From year 2, onward, the function showed the same global shape as described in year 5 (data not shown). Also, it is noticeable that, in year 5, the response function to BA had an almost identical e.d.f. value to that of IL, even though the two functions had different curve shapes. Finally, the TSD curve was almost a horizontal line on the *x*-axis, hence having a negligible effect on STAR values throughout the input parameter domain. The e.d.f. values for TSD showed small fluctuations and values close to 1, from 1·14 to 1·51, throughout the 5 years of tree growth.

#### Topology

The prediction of STAR values with the GAM model was performed for two specific combinations of input parameter values. At the first step, the number of proleptic and sylleptic laterals per unit length was considered (Table 3). In the first year of growth, the approximate effects on STAR revealed that only the sylleptic density had a significant influence. From year 2 to year 5, both sylleptic and proleptic densities showed a significant effect on the STAR values, with a marked higher influence from the sylleptic density each year (Table 3). The estimated GAM explained 62·3% in year 1 to 31% in year 5 of the total variation of STAR values obtained from simulations (Table 3, adjusted *R*^2^).

**Table 3.** Approximate effects of the topological input parameters on STAR values with a GAM

Year	Input variable	e.d.f.	*F*	*P*-value	*R*^2^
1	syll l^–1^	3·23	38·04	<2 × 10^–16^***	0·6
	pro l^–1^	2·98	1·73	0·15	
2	syll l^–1^	1·05	35·38	2·17 × 10^–8^***	0·42
	pro l^–1^	3·45	8·59	1·51 × 10^–5^***	
3	syll l^–1^	2·44	3·09	2·05 × 10^–4^***	0·31
	pro l^–1^	4·16	5·13	6·84 × 10^–4^***	
4	syll l^–1^	2·27	2·870	2·26 × 10^–5^***	0·35
	pro l^–1^	4·58	5·612	2·37 × 10^–4^***	
5	syll l^–1^	2·05	2·593	1·57 × 10^–5^***	0·31
	pro l^–1^	4·17	5·142	1·69 × 10^–3^**	

Input variables were the number of sylleptic (syll) and proleptic (pro) shoots per unit length (l^–1^).

*** *P* ≤ 0·001; **P ≤ 0·01.

**Fig. 6.**
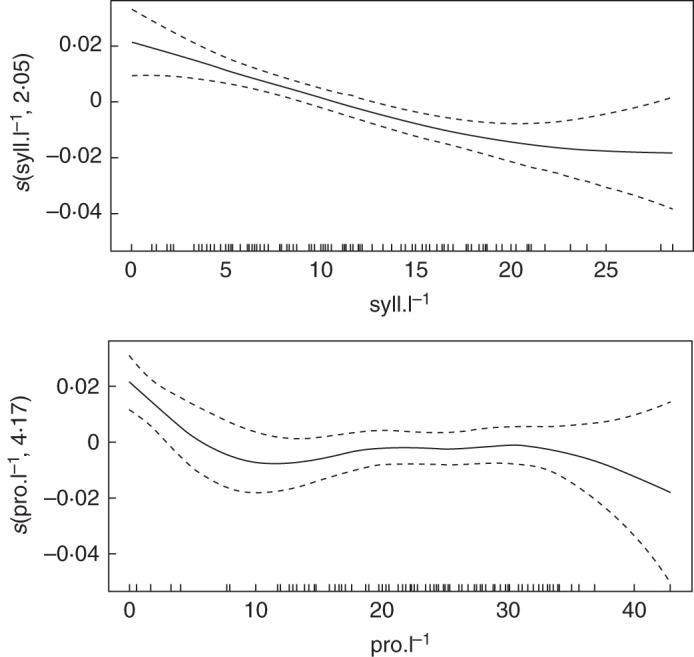
Expected mean values (solid lines) and confidence band (dashed lines) of smoothed terms (*y*-axes: predicted STAR value) depending on the input parameter values in the topology experiment (*x*-axes: position of sampled values) estimated from the generalized additive model (GAM). The term *s* in the labels on the *y*-axes indicates that thin plate splines are used, and the number corresponds to the equivalent degree of freedom (e.d.f.) of the estimated curve.

An increasing density of sylleptic laterals had a negative impact on the expected mean STAR value, as shown in Fig. 6 (left) for the fifth year. In that year, the negative effect on STAR of the increasing sylleptic density was monotonous and stronger until 20, where it seemed to reach a stable influence. However, the confidence interval became important after this value because of the insufficient number of samples. The response function had a similar shape every year except in year 2 (data not shown) where the decrease was linear all along as shown by the e.d.f. values close to 1. The function of STAR responses to proleptic density showed a more complex shape with more fluctuations (Fig. 6, right). It started with a negative effect of an increased density of proleptic laterals on mean STAR values, up to about 10, at which point it became slightly positive until 20. From 20 to about 30, the influence of proleptic density was stable and then reverted to a negative effect similar to that observed before 10. However, after 35, the confidence interval also became important for the same reason of insufficient sampling. In the case of proleptic laterals, the evolution of the response function toward the shape described in year 5 started in year 2. In year 1, the response function was a horizontal flat curve around 0, illustrating the absence of influence of proleptics in that year shown in Table 3.

In a second step, the density of each type of sylleptic and proleptic (long, medium and short) was considered (Table 4).

**Table 4.** Approximate effects of the topological input parameters on STAR values with a GAM

Year	Input variable	e.d.f.	*F*	*P*-value	*R*^2^
1	sl l^–1^	3·51	12·53	1·31 × 10^–8^***	0·58
	sm l^–1^	2·13	7·98	1·68 × 10^–4^***	
	ss l^–1^	1·00	2·55	0·113833	
	pl l^–1^	1·00	3·66	0·058766	
	pm l^–1^	1·00	1·83	0·178819	
	ps l^–1^	1·92	3·01	0·044906*	
2	sl l^–1^	2·34	16·85	8·87 × 10^–9^***	0·57
	sm l^–1^	1·00	11·44	0·00104**	
	ss l^–1^	1·00	0·26	0·61205	
	pl l^–1^	1·36	0·19	0·77948	
	pm l^–1^	3·14	7·82	1·97 × 10^–5^***	
	ps l^–1^	1·20	0·04	0·90516	
3	sl l^–1^	2·15	20·61	1·4 × 10^–9^***	0·54
	sm l^–1^	1·84	1·77	0·17187	
	ss l^–1^	6·31	1·06	0·39947	
	pl l^–1^	4·02	0·99	0·42864	
	pm l^–1^	3·96	3·96	0·00307**	
	ps l^–1^	1·57	0·57	0·55967	
4	sl l^–1^	2·20	20·32	8·54 × 10^–10^***	0·53
	sm l^–1^	1·82	2·71	0·0649	
	ss l^–1^	1·00	1·66	0·2008	
	pl l^–1^	1·00	1·19	0·2773	
	pm l^–1^	3·99	3·06	0·0138*	
	ps l^–1^	1·00	0·001	0·9780	
5	sl l^–1^	1·90	21·32	2·22 × 10^–9^***	0·42
	sm l^–1^	1·00	1·72	0·1927	
	ss l^–1^	1·00	0·002	0·9652	
	pl l^–1^	2·56	1·28	0·2869	
	pm l^–1^	1·37	3·63	0·0386*	
	ps l^–1^	1·00	0·000	0·9877	

Input variables were the number of types of sylleptic and proleptic shoots, long (sl and pl), medium (sm and pm) and short (ss and ps), respectively, per unit length (l^–1^).

*** *P* ≤ 0·001; **P ≤ 0·01; *P ≤ 0·05.

In the first two years, both long and medium sylleptic densities had significant effects, and long sylleptic density remained significant in the subsequent years (Table 4). Among the three types of proleptic shoots, the highest impact was obtained for the density of medium shoots whose effect was significant from the second to the fifth year. However, this effect was much less compared with that of medium sylleptic density, and it also decreased in years 4 and 5 (see *F*-values, Table 4) The short proleptic density had a significant effect in the first year only, and the long proleptic density had no effect. The estimated GAM explained 58% of the total variation of STAR values in the first year of growth and decreased to 41·8% in year 5 (Table 4, adjusted *R*^2^). If we were to decompose this GAM into two models, for the density of long, medium and short sylleptic or proleptic shoots, respectively, the model with syllpetic shoots would explain 40–50% of the STAR variation over the years whereas only 10% would be explained by the density of proleptic shoots (data not shown). The combination of the two types of laterals in a single model thus explained the STAR values more , in an additive way.

**Fig. 7.**
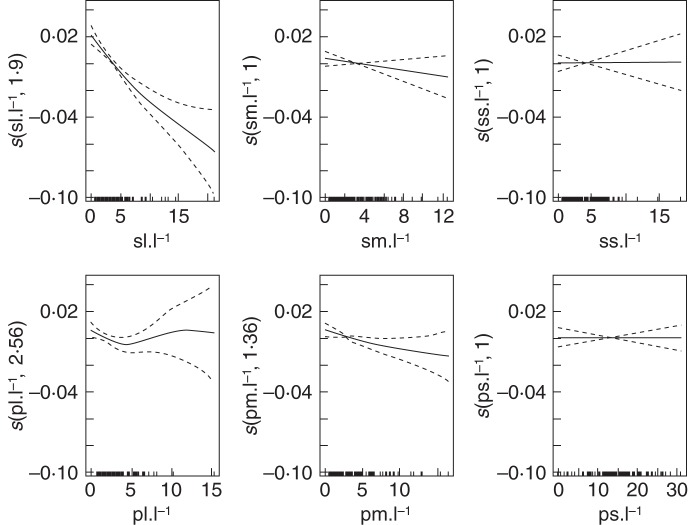
Expected mean values (solid lines) and confidence band (dashed lines) of smoothed terms (*y*-axes: predicted STAR value) depending on the input parameter values in the topology experiment, with a decomposition of lateral types into length categories (*x*-axes: position of sampled values) estimated from the generalized additive model (GAM). The term *s* in the labels on the *y*-axes indicates that thin -plate splines are used, and the number corresponds to the equivalent degree of freedom (e.d.f.) of the estimated curve.

An increasing long sylleptic density had a negative effect on the expected STAR values, as shown in Fig. 7, for the fifth year. In that year, the negative effect on STAR of the increasing long sylleptic density was rapid and monotonous, with a stronger decrease until 8 where its influence slightly diminished. However, the confidence interval became important after this value because of the insufficient number of samples. The response function had a similar shape in all years (data not shown). The negative effect on STAR of the increasing medium proleptic density was similar but much milder than that of the long sylleptic density. In year 5, the negative effect was almost linear, but with a large interval confidence when the density was >8 (Fig. 7). The shape of the response function from year 2 to year 4 had more marked steps than that in year 5. They started with a negative effect up to 4 and then reached a zone of stable influence from 4 to 12 before reverting to the negative effect from 12 onward (data not shown). It must be noted that the slight effect of short proleptic density in year 1 corresponded to a response function with a positive effect on the STAR value from 0 to 10 and almost no effect from there onwards (data not shown). All other response functions barely changed over the years, except for year 3, and were close to a horizontal straight line near the *x*-axis (data not shown). Therefore the e.d.f. values were close to 1. In year 3, the response functions for both the short sylleptic and long proleptic densities exhibited strong fluctuations around the *x*-axis, as shown by the high e.d.f. values of 6·31 and 4·02, respectively.

## DISCUSSION

The *in silico* experiments performed in the present study, by varying either organ geometry or branching traits in the first year of tree development, generated a large range of tree architectures with different TLA and STAR values. The important difference in TLA between the geometry and topology experiments revealed that branching along the first annual shoot of the trunks enhanced tree architecture variability and variance in TLA. The mean values of TLA simulated for 5-year-old trees appeared high in comparison with values obtained on digitized and pruned trees (Willaume *et al.*, 2004). However, this is not surprising if we consider the large range of input values that were used for the simulations. The evolution over the years of the ratio between the mean TLA values for the two experiments seemed reasonable given the fact that the trees in the geometry experiment did not have sylleptic shoots in year 1. However, the outlier individuals in the topology experiment, which again exhibited a TLA 3–5 times higher than the mean value, are probably unrealistic model output. Among the reasons that may be involved, the absence of shoot mortality regulation in the MAppleT model is probably a major factor. Indeed, in highly branched young trees, strong competition between shoots may lead to high mortality rates, in particular in the very centre of a very dense canopy. Different factors such as correlation inhibition (Umeki *et al.*, 2006), fruiting (Lampinen *et al.*, 2010) and light transmittance (Takenaka, 2000) are likely to be responsible for insufficient shoot growth and a natural mortality. Presently, in the model, mortality rate is constant and is of concern for short shoots only (Costes *et al.*, 2008). As a consequence, all the long and medium shoots are fully grown and therefore participate in both the TLA increase and the STAR reduction.

Whatever the input parameters, the mean STAR values estimated on simulated trees decreased with the year of growth with a stable variability in both experiments. On the one hand, these patterns resulted from the low overlap between leaves in young trees and from the increase in the mean number of leaves and GUs that increased with the tree age and were responsible for the TLA increase (Han *et al.*, 2012). On the other hand, the predominance of short shoots in within-tree shoot demography when coming to full maturity (Costes *et al.*, 2003) also contributed to the STAR decrease. A previous study on the relationship between the within-tree organization at different scales and LIE showed that the shoot scale was responsible for most of the tree foliage aggregation and thus foliage overlap (Da Silva *et al.*, 2014). The stabilization of STAR values in year 4 and 5 was also observed in that study and was related to a strong organizational development that is taking place when the shoot scale becomes clearly distinct from the branch scale, i.e. in years 2 and 3. Compared with the STAR values of that study, our results from the geometry experiment were in accordance for the mean but with a substantially higher variability. In contrast, the topology experiment exhibited a similar variability but slightly lower mean STAR values than in Da Silva *et al.* (2014). The lower mean values of the topology experiment are the direct consequence of the TLA excess as previously stated. Also, the mean values of STAR obtained in 5-year-old trees were underestimated with respect to previous estimations on digitized trees (Willaume *et al.*, 2004; Massonnet *et al.*, 2008). As stated previously for TLA, these discrepancies are likely to result from the large range of values we explored which do not necessarily match values observed in particular cultivars.

The low STAR variability in the topology experiment despite the high variance of TLA, which is the STAR normalizing term, suggests a threshold in terms of LIE that is not overcome by the sole increase of leaf area. Comparing the results of the two experiments highlights a higher impact on STAR when varying organ geometry than early branching. This may be due to the repeated occurrence of individual organs over the years whereas the topology has a declining effect linked to tree ontogeny.

The sensitivity analysis performed in the present study allowed us to highlight a hierarchy among the aspects that were expected to have a direct influence on the STAR value of the whole tree. Among the organ geometrical traits that were considered, internode length and area of individual leaves had the highest impact on STAR, whereas the branching angle had only a low impact. Top shoot diameter had a very low if not null impact. An individual leaf area effect is consistent with the definition of STAR (Oker-Blom and Smolander, 1988). Basically, the overall STAR turned out to be smaller when the leaf area was higher because large leaves produce larger overlaps (independently of possible leaf blade reorientation toward the light source), thereby less light can reach the inner leaves. Moreover, large leaves also mean more biomass and therefore more bending of branches which bring more leaves under the overlaps (independently of the relatively larger effect of fruit biomass on branch bending). The internode effect on STAR is likely to be less direct than that of leaf area. As previously, two main reasons may explain this strong influence: internodes are the primary support minimizing leaf overlap, and it will influence the branch bending (Alméras *et al.*, 2004; Moulia and Fournier, 2009). The fact that internode length had the highest impact on STAR values is consistent with previous studies which have underlined the large influence of internode length on plant light interception in other plant species (Christophe *et al.*,2003; Pearcy *et al.*, 2005; Sarlikioti *et al.*, 2011). However, internode length also depends on the local light environment during its development (Kahlen and Stützel, 2011), and more complex relationships between organ geometry and environmental conditions could be considered in the simulations.

Among branching traits, long sylleptic shoots had the highest impact and this impact persisted over the years. This is consistent with the use of sylleptic branching (also called feathers by horticulturists) in initial tree training, in particular for promoting earlier fruiting (Wertheim, 1978; Costes *et al.*, 2006). The present study highlights possible counterproductive effects of the presence of sylleptic shoots along the trunks by reducing the light interception in subsequent years. This negative effect is usually overcome by a careful choice of lateral branches along the trunks during tree training (Forshey *et al.*, 1992). However these manual interventions in the trees are expensive in terms of time and labour. Therefore, from the present study we can assume that breeding and genetic selection could open up a new avenue for light interception optimization, and could be more efficient for saving time, labour and resources than yearly topology alteration through pruning. However, it must be remembered that sylleptic branching is usually assumed to be involved in tree plasticity in response to environmental conditions (Wu and Hinckley, 2001). Even though the number of sylleptic laterals has been shown to be highly heritable in the first year of tree growth (Segura *et al.*, 2008), a better understanding of the interactions between genetic and environmental effects would help in designing varietal ideotypes. In contrast and contrary to our expectations, long proleptic shoots had a lower impact on STAR than medium proleptic shoots. This may be due to their low number within the trees (Costes and Guédon, 1997). Indeed these laterals are strongly linked to acrotony (Cline, 1997) and occur on a few nodes only, just below the apical bud. In our opinion, the analysis of the impact of genetically controlled traits on response factors such as LIE, in the case of an isolated tree, was a first necessary step before considering the case of a tree in orchard conditions. Indeed, in that case, there are many new factors that would be necessary to be taken into account such as the effects of site latitude, planting density, row width and azimuth. This additional complexity will introduce noise on the response factors due to the agronomic aspects that can only be separated from the the genetic aspects if their impacts are already known and understood.

From a methodological point of view, the GAM modelling provided estimations of STAR variations with *R*^2^ >95% in the geometry experiment. The additive effects of the four factors allowed us to estimate the contributions of each factor to the variation of STAR, and the STAR values could be predicted with good confidence from a simple combination of functions built with leaf area, internode length and branching angle. However, confidence in the model outputs could be related to the range of variation in the input parameters we have explored, and the validity of model predictions with another range of input variables values will have to be further investigated. The predictions of STAR values with GAM were less accurate in the topology experiment, due to lower *R*^2^ values and more complex response functions. Indeed, if the effect of sylleptic density was relatively simple, the response function to proleptic density was more complex, with effect depending on the *x*-axis values. The low values of adjusted *R*^2^ partly result from the fact that the GAM procedure did not allow us to estimate high dimensional smooth functions taking into account both main and interaction effects between parameters, on a data set limited to 100 points. Even though it was not possible to enrich our data set on the topology study with many additional simulations, due to simulation costs, some additional computations were performed (unpubl. data) which showed that interactions do not have null but have moderate contributions to the variation in STAR values. Therefore, most of the unexplained variations are due to the stochastic component of the MAppleT model and to the low effect of branching density parameters on STAR values after the second year. It must be noted that the chosen descriptors of the initial tree topology, i.e. branching density, only partly represent the complex branching patterns along trunks, and this may contribute to their low effect on STAR.

In the case of the geometry experiment, we knew from a previous study that the four studied factors had mainly additive effects (Han *et al.*, 2012). However, in the case of the topological experiment, we cannot reject the hypothesis of an interaction because the data set size allowed us to estimate the main effects only. Also, the dependency between lateral types on the total number of nodes of a parent shoot, the strong branching organization patterns observed along shoots (Guédon *et al.*, 2001; Renton *et al.*, 2006) as well as the mutual exclusion of sylleptic and proleptic categories at a given node suggest that these input variables are dependent. Further studies using model selection (including or not interaction terms) in generalized additive modelling (Wood, 2006) would certainly allow a more refined analysis. Moreover, in the topology experiment, the function response led us to suspect that the sylleptic and proleptic shoots densities (including long, medium and short) have conditional effects, i.e. a parameter having an effect only when the first one considered is in a certain range. From this perspective, other statistical approaches could be explored to model the response of STAR, such as the recursive partitioning described in Storlie and Helton (2008) or using Gaussian process modelling, such as Marrel *et al.* (2012). It would also be possible to extend the GAM in a multidimensional way to combine the different input parameters considered, such as for instance IL and LA in the geometry experiment or even combining geometrical and topological parameters. However, this would require more numerous simulated data to obtain estimators of good quality and more complex designs of the experimental plan for simulations. Considering the relatively long time required for simulating each tree over 5 years, more complex plans will certainly require coupling an optimization process to the sensitivity analysis (Picheny *et al.*, 2013).

Finally, this study appeared very promising for identifying traits that could control light interception optimization, from the breeders point of view. Indeed, it suggests that major genetic improvements could be made in fruit tree species, as previously performed in annual crops (Khush, 2001). Nevertheless, the search for innovative ideotypes could also benefit from a focus on the 1 or 2% of unexplained variations which may lead to less expected effects. Presently, a major limitation of our approach results from the fact that the *in silico* experimentation design did not include constraints between input parameters. For instance, the simulated trees can exhibit a very small leaf area combined with very long internodes, such combinations leading to the highest STAR values at the whole-tree level. Nevertheless, in reality, it is unlikely that the leaf area changes independently from the other factors, especially internode length, due to strong allometric properties of the metamers; see, for example, Yang *et al.* (2010). As a consequence, the definition of the input parameter space to be explored in the sensitivity analysis would need further investigations. Similarly the way to balance the different and possibly antagonist functions of plant organs will have to be considered to develop our approach further towards the concept of optimal design and ideotype definition (Wu, 1998). For instance, even though an increase in branching density negatively impacts light interception within the tree, it may also have a positive impact on the tree fruiting potential, by multiplying the number of fructification locations. Also, the individual leaf areas which impact light interception are involved in transpiration and photosynthesis, and whereas small leaves decrease their overlap and increase STAR, large leaves are favourable as sources of carbohydrates. From this perspective, other output variables should be combined with light interception, considering physiological (e.g. photosynthesis) or agronomic (e.g. production) traits simultaneously in a multiobjective approach as recently performed by Quilot *et al.* (2013) on peach fruit quality and sensitivity to brown rot. Presently, using a single output variable makes the prediction of the best combination of traits to be considered for selecting an optimal apple tree architecture hazardous. The use of *in silico* scenarios and optimization procedures should allow us in the future to account for antagonistic effects of input parameters, possibly at several scales of tree organization, and their combined effects on multiple objectives.

## References

[MCU034C1] Alméras T, Gril J, Costes E (2002). Bending of apricot-tree branches under the weight of axillary productions: test of a mechanical model with experimental data. Trees.

[MCU034C2] Alméras T, Costes E, Salles JC (2004). Identification of biomechanical factors involved in stem form variability between apricot tree varieties. Annals of Botany.

[MCU034C3] Andrivon D, Giorgetti C, Baranger A (2013). Defining and designing plant architectural ideotypes to control epidemics. European Journal of Plant Pathology.

[MCU034C4] Barthélémy D, Caraglio Y (2007). Plant architecture: a dynamic, multilevel and comprehensive approach to plant form, structure and ontogeny. Annals of Botany.

[MCU034C5] Bell DL, Galloway LF (2008). Population differentiation for plasticity to light in an annual herb: adaptation and cost. American Journal of Botany.

[MCU034C6] Boudon F, Pradal C, Cokelaer T, Prusinkiewicz P, Godin C (2012). L-Py: an L-System simulation framework for modeling plant development based on a dynamic language. Frontiers in Plant Science.

[MCU034C7] Brown LK, George TS, Dupuy LX, White PJ (2013). A conceptual model of root hair ideotypes for future agricultural environments: what combination of traits should be targeted to cope with limited P availability?. Annals of Botany.

[MCU034C8] Byrne M, Murrell JC, Owen JV, Kriedemann P, Williams ER, Moran GF (1996). Identification and mode of action of quantitative trait loci affecting seedling height and leaf area in Eucalyptus nitens. Theoretical and Applied Genetics.

[MCU034C9] Cairns JE, Sanchez C, Vargas M, Ordonez R, Araus JL (2012). Dissecting maize productivity: ideotypes associated with grain yield under drought stress and well-watered conditions. Journal of Integrative Plant Biology.

[MCU034C10] Chardon F, Noel V, Masclaux-Daubresse C (2012). Exploring NUE in crops and in *Arabidopsis* ideotypes to improve yield and seed quality. Journal of Experimental Botany.

[MCU034C11] Christophe A, Moulia B, Varlet-Grancher C (2003). A quantitative analysis of the three-dimensional spatial colonization by a plant as illustrated by white clover (*Trifolium repens* L.). International Journal of Plant Sciences.

[MCU034C12] Cline MG (1997). Concepts and terminology of apical dominance. American Journal of Botany.

[MCU034C13] Côté J-F, Widlowski J-L, Fournier RA, Verstraete MM (2009). The structural and radiative consistency of three-dimensional tree reconstructions from terrestrial lidar. Remote Sensing of Environment.

[MCU034C14] Costa P, Durel CE (1996). Time trends in genetic control over height and diameter in maritime pine. Canadian Journal of Forest Research.

[MCU034C15] Costes E, Guédon Y (1997). Modeling the sylleptic branching on one-year-old trunks of apple cultivars. Journal of the American Society for Horticultural Science.

[MCU034C16] Costes E, Guedon Y (2002). Modelling branching patterns on 1-year-old trunks of six apple cultivars. Annals of Botany.

[MCU034C17] Costes E, Guédon Y (2012). Deciphering the ontogeny of a sympodial tree. Trees – Structure and Function.

[MCU034C18] Costes E, Sinoquet H, Kelner JJ, Godin C (2003). Exploring within-tree architectural development of two apple tree cultivars over 6 years. Annals of Botany.

[MCU034C19] Costes E, Lauri PE, Régnard JL (2006). Tree architecture and production. Horticultural Reviews.

[MCU034C20] Costes E, Smith C, Renton M, Guédon Y, Prusinkiewicz P, Godin C (2008). MAppleT: simulation of apple tree development using mixed stochastic and biomechanical models. Functional Plant Biology.

[MCU034C21] Da Silva D, Boudon F, Godin C, Sinoquet H (2008). Multiscale framework for modeling and analyzing light interception by trees. Multiscale Modeling and Simulation.

[MCU034C22] Da Silva D, Han L, Costes E (2014). Light interception efficiency of apple trees: a multiscale computational study based on MappleT.

[MCU034C23] Dechaine JM, Johnston JA, Brock MT, Weinig CW (2007). Constraints on the evolution of adaptive plasticity: costs of plasticity to density are expressed in segregating progenies. New Phytologist.

[MCU034C24] Den Dulk JA (1989). The interpretation of remote sensing, a feasibility study.

[MCU034C25] Degasperi A, Gilmore S (2008). Sensitivity analysis of stochastic models of bistable biochemical reactions. Lecture Notes in Computer Science.

[MCU034C26] Dickman DI, Gold MA, Fore JA (1994). The ideotype concept and the genetic improvement of tree crops. Plant Breeding Review.

[MCU034C27] Donald CM (1968). The breeding of crop ideotypes. Euphytica.

[MCU034C28] Durel CE, Laurens F, Fouillet, Lespinasse Y (1998). Utilization of pedigree information to estimate genetic parameters from large unbalanced data sets in apple. Theoretical and Applied Genetics.

[MCU034C29] Faivre R, Iooss B, Mahévas S, Makowski D, Monod H (2013). Analyse de sensibilité et exploration de modèles. Applications aux modèles environnementaux.

[MCU034C30] Forshey CG, Elfving DC, Stebbins L (1992). Training and pruning apple and pear trees.

[MCU034C31] Guédon Y, Barthélémy D, Caraglio Y, Costes E (2001). Pattern analysis in branching and axillary flowering sequences. Journal of Theoretical Biology.

[MCU034C32] Han L, Da Silva D, Boudon F (2012). Investigating the influence of geometrical traits on light interception efficiency of apple trees: a modelling study with MAppleT. International Symposium on Plant Growth Modeling, Simulation, Visualization and Applications.

[MCU034C33] Kahlen K, Stützel H (2011). Modelling photo-modulated internode elongation in growing glasshouse cucumber canopies. New Phytologist.

[MCU034C34] Khush GS (2001). Green revolution: the way forward. Nature Reviews Genetics.

[MCU034C35] van Kleunen M, Fischer M (2004). Constraints on the evolution of adaptive phenotypic plasticity in plants. New Phytologist.

[MCU034C36] Lampinen BD, Tombesi S, Metcalf SG, DeJong TM (2010). Spur behaviour in almond trees: relationships between previous year spur leaf area, fruit bearing and mortality. Tree Physiology.

[MCU034C37] Laurens F, Audergon JM, Claverie J (2000). Integration of architectural types in French programmes of ligneous fruit species genetic improvement. Fruits (Paris).

[MCU034C38] Lauri PE (2002). From tree architecture to tree training – an overview of recent concepts developed in apple in France. Journal of the Korean Society for Horticultural Sciences.

[MCU034C39] Lauri PE, Costes E (2004). Progress in whole-tree architectural studies for apple cultivar characterization at INRA, France – contribution to the ideotype approach. Acta Horticulturae.

[MCU034C40] Le May C, Ney B, Lemarchand E, Schoeny A, Tivoli B (2009). Effect of pea plant architecture on spatiotemporal epidemic development of ascochyta blight (*Mycosphaerella pinodes*) in the field. Plant Pathology.

[MCU034C41] Lespinasse JM (1977). La conduite du Pommier. I – Types de fructification. Incidence sur la conduite de l'arbre.

[MCU034C42] Lespinasse Y (1992). Breeding apple tree: aims and methods. Proceedings of the Joint Conference of the EAPR Breeding and Varietal Assessment Section and the EUCARPIA Potato Section.

[MCU034C43] Livny Y, Yan F, Olson M, Chen B, Zhang H, El-Sana J (2010). Automatic reconstruction of tree skeletal structures from point clouds. ACM Transaction on Graphics.

[MCU034C44] Lurette A, Touzeau S, Lamboni M, Monod H (2009). Sensitivity analysis to identify key parameters influencing Salmonella infection dynamics in a pig batch. *Journal of Theoretical Biol*ogy.

[MCU034C45] Marrel A, Iooss B, Da Veiga S, Ribatet M (2012). Global sensitivity analysis of stochastic computer models with joint metamodels. Statistics and Computing.

[MCU034C46] Massonnet C, Regnard JL, Lauri PE, Costes E, Sinoquet H (2008). Contributions of foliage distribution and leaf functions to light interception, transpiration and photosynthetic capacities in two apple cultivars at branch and tree scales. Tree Physiology.

[MCU034C47] Moon P, Spencer DE (1942). Illumination from a non-uniform sky.

[MCU034C48] Moulia B, Fournier M (2009). The power and control of gravitropic movements in plants: a biomechanical and systems biology view. Journal of Experimental Botany.

[MCU034C49] Oker-Blom P, Smolander H (1988). The ratio of shoot silhouette area to total needle area in Scots pine. Forest Science.

[MCU034C50] Oraguzie NC, Hofstee ME, Brewer LR, Howard C (2001). Estimation of genetic parameters in a recurrent selection program in apple. Euphytica.

[MCU034C51] Pearcy RW, Muraoka H, Valladares F (2005). Crown architecture in sun and shade environments: assessing function and trade-offs with a three-dimensional simulation model. New Phytologist.

[MCU034C52] Peng S, Khush GS, Virk P, Tang Q, Zou Y (2008). Progress in ideotype breeding to increase rice yield potential. Field Crops Research.

[MCU034C53] Picheny V, Ginsbourger D (2013). Noisy kriging-based optimization methods: a unified implementation within the DiceOptim package. Computational Statistics and Data Analysis.

[MCU034C54] Pradal C, Dufour-Kowalski S, Boudon F, Fournier C, Godin C (2008). OpenAlea: a visual programming and component-based software platform for plant modelling. Functional Plant Biology.

[MCU034C55] Preuksakarn C, Boudon F, Ferraro P, Durand J-B, Nikinmaa E, Godin C (2010). Reconstructing plant architecture from 3D laser scanner data. Proceedings of the 6th International Workshop on Functional–Structural Plant Models.

[MCU034C56] Quilot-Turiona B, Ould-Sidib MM, Kadranib A, Hilgert N, Génard M, Lescourret F (2013). Optimization of parameters of the ‘Virtual Fruit’ model to design peach genotype for sustainable production systems. European Journal of Agronomy.

[MCU034C57] Raumonen P, Kaasalainen M, Åkerblom M (2013). Fast automatic precision tree models from terrestrial laser scanner data. Remote Sensing.

[MCU034C58] Renton M, Guédon Y, Godin C, Costes E (2006). Similarities and gradients in growth unit branching patterns during ontogeny in *Fuji* apple trees: a stochastic approach. Journal of Experimental Botany.

[MCU034C59] Richards RA, Rebetzke GJ, Condon AG, van Herwaarden AF (2002). Breeding opportunities for increasing the efficiency of water use and crop yield in temperate cereals. Crop Science.

[MCU034C60] Ripetti V, Escoute J, Verdeil J, Costes E (2008). Shaping the shoot: the relative contribution of number of cells and cell size in the variation of internode length between parent and hybrids apple trees. Journal of Experimental Botany.

[MCU034C61] Salamini F (2003). Hormones and the green revolution. Science.

[MCU034C62] Saltelli A, Chan K, Scott EM (2000). Sensitivity analysis: gauging the worth of scientific models.

[MCU034C63] Sarlikioti V, de Visser PHB, Buck-Sorlin GH, Marcelis LFM (2011). How plant architecture affects light absorption and photosynthesis in tomato: towards an ideotype for plant architecture using a functional–structural plant model. Annals of Botany.

[MCU034C64] Saudreau M, Marquier A, Adam B, Sinoquet H (2011). Modelling fruit-temperature dynamics within apple tree crowns using virtual plants. Annals of Botany.

[MCU034C65] Segura V, Cilas C, Laurens F, Costes E (2006). Phenotyping progenies for complex architectural traits: a strategy for 1-year-old apple trees (*Malus×domestica* Borkh.). Tree Genetics and Genomes.

[MCU034C66] Segura V, Denancé C, Durel CE, Costes E (2007). Wide range QTL analysis for complex architectural traits in a 1-year-old apple progeny. Genome.

[MCU034C67] Segura V, Cilas C, Costes E (2008). Dissecting apple tree architecture into genetic, ontogenetic and environmental effects: mixed linear modelling of repeated spatial and temporal measures. New Phytologist.

[MCU034C68] Segura V, Durel C-E, Costes E (2009). Dissecting apple tree architecture into genetic, ontogenetic and environmental effects: QTL mapping. Tree Genetics and Genomes.

[MCU034C69] Shinozaki K, Yoda K, Hozumi K, Kira T (1964). A quantitative analysis of plant form – the pipe model theory: I. basic analyses. Japanese Journal of Ecology.

[MCU034C70] Sinoquet H, Moulia B, Bonhomme R (1991). Estimating the three-dimensional geometry of a maize crop as an input of radiation models: comparison between three-dimensional digitizing and plant profiles. Agricultural and Forest Meteorology.

[MCU034C71] Sinoquet H, Rivet P (1997). Measurement and visualization of the architecture of an adult tree based on a three-dimensional digitising device. Trees – Structure and Function.

[MCU034C72] Stenberg P (1996). Simulations of the effects of shoot structure and orientation on vertical gradients in intercepted light by conifer canopies. Tree Physiology.

[MCU034C73] Storlie CB, Helton JC (2008). Multiple predictor smoothing methods for sensitivity analysis: description of techniques. Reliability Engineering and System Safety.

[MCU034C74] Takenaka A (2000). Shoot growth responses to light microenvironment and correlative inhibition in tree seedlings under a forest canopy. Tree Physiology.

[MCU034C75] Tancred SJ, Zeppa AG, Cooper M, Stringer JK (1995). Heritability and patterns of inheritance of the ripening date of apples. HortScience.

[MCU034C76] Taylor-Hell J (2005). Biomechanics in botanical trees.

[MCU034C77] Thomas RG, Hay MJM (2008). Regulation of shoot branching patterns by the basal root system: towards a predictive model. Journal of Experimental Botany.

[MCU034C78] Umeki K, Seino T, Lim E, Honjo T (2006). Patterns of shoot mortality in Betula platyphylla in northern Japan. Tree Physiology.

[MCU034C79] Villa-Vialaneix N, Follador M, Ratto M, Leip A (2012). A comparison of eight metamodeling techniques for the simulation of N2O fluxes and N leaching from corn crops. Environmental Modelling and Software.

[MCU034C80] Wertheim SJ (1978). Manual and chemical induction of side-shoot formation in apple trees in the nursery. Scientia Horticulturae.

[MCU034C81] Willaume M, Lauri PE, Sinoquet H (2004). Light interception in apple trees influenced by canopy architecture manipulation. Trees – Structure and Function.

[MCU034C82] Wood S (2006). Generalized additive models: an introduction with R, Vol. 66.

[MCU034C83] Wu RL (1998). Genetic mapping of QTLs affecting tree growth and architecture in *Populus*: implication for ideotype breeding. Theoretical and Applied Genetics.

[MCU034C84] Wu Q, Cournède P-H, Mathieu A (2012). An efficient computational method for global sensitivity analysis and its application to tree growth modelling. Reliability Engineering and System Safety.

[MCU034C85] Wu R, Hinckley TM (2001). Phenotyping plasticity of sylleptic branching: genetic design of tree architecture. Critical Reviews in Plant Sciences.

[MCU034C86] Wünsche JN, Lakso AN (2000). The relationship between leaf area and light interception by spur and extension shoot leaves and apple orchard productivity. HortScience.

[MCU034C87] Yang D, Niklas KJ, Xiang S, Sun S (2010). Size-dependent leaf area ratio in plant twigs: implication for leaf size optimization. Annals of Botany.

